# Supplementary material for: Weil’s Disease in an HIV-Infected Patient: A Case Report and Literature Review
Source: Diagnostics (Basel). 2023 Oct 16;13(20):3218. doi: 10.3390/diagnostics13203218 (PMC10606346; doi:10.3390/diagnostics13203218)
Supplement: Supplementary file 1 [file diagnostics-13-03218-s001.zip › diagnostics-2626955-supplementary.pdf]

Tittle: Weil's disease in a HIV-infected patient: a case report and literature review

Xinchun Zheng[1†], Pengyuan He[1†], Ruihua Zhong[1], Gongqi Chen[1], Jinyu Xia[1*], Chunna Li[1*]

[1] Infectious Disease Prevention and Treatment Center, the Fifth Affiliated Hospital of Sun Yat-Sen University, Zhuhai 519000, Guangdong, China

Xinchun Zheng e-mail: zhengxch8@mail.sysu.edu.cn

Pengyuan He e-mail: hepy@mail.sysu.edu.cn

Ruihua Zhong e-mail: zhongrh8@mail.sysu.edu.cn

Gongqi Chen e-mail: Haizi2308@sina.com

Jinyu Xia e-mail: xiajinyu@mail.sysu.edu.cn

Chunna Li e-mail: lichunna@mail.sysu.edu.cn

[†] Xinchun Zheng and Pengyuan He contributed equally to this manuscript.

[*] Chunna Li and Jinyu Xia are the co-corresponding authors to this manuscript.

Correspondence: Chunna Li, the Fifth Affiliated Hospital of Sun Yat-Sen University, Zhuhai 519000, Guangdong, China (lichunna@mail.sysu.edu.cn);

Jinyu Xia, the Fifth Affiliated Hospital of Sun Yat-Sen University, Zhuhai 519000, Guangdong, China (xiajinyu@mail.sysu.edu.cn).

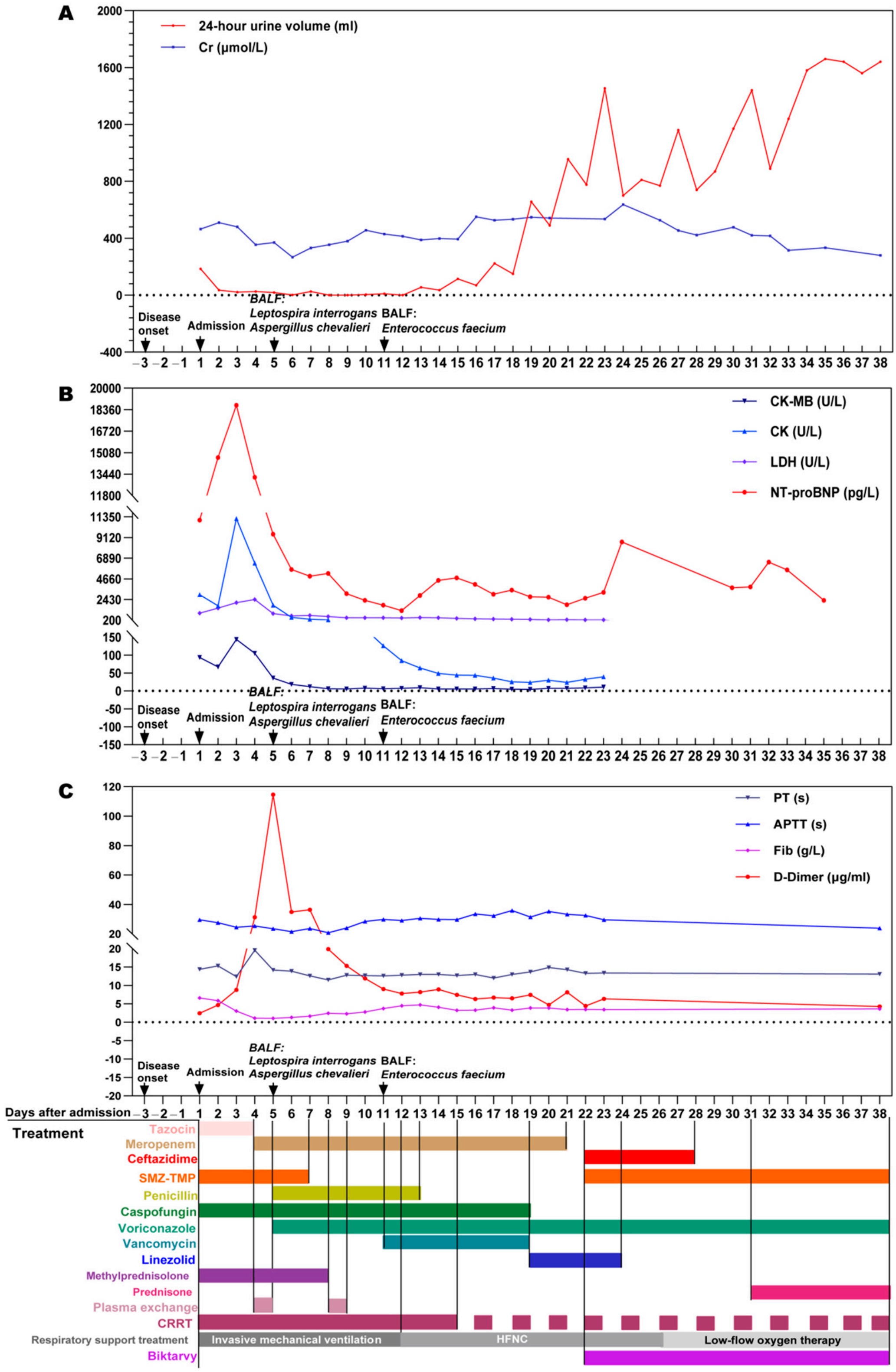

Figure S1 Treatment process and dynamic changes of clinical test parameters of the patient in the clinical course. Dynamic change of creatinine and 24-hour urine volume (Panel A), cardiac enzymes (CK-MB, CK, HDL) and cardiac function indicators (pro-BNP) (Panel B), coagulation indicators (PT, APTT, Fib, D-Dimer) (Panel C) during hospitalization.

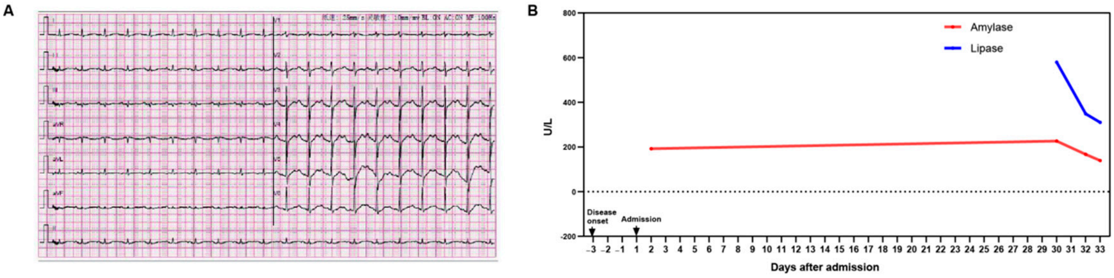

Figure S2 The ECG showed rapid atrial fibrillation on 2 days after admission (Panel A); The dynamic changes in lipase and amylase levels were showed during hospitalization (Panel B).
